# Identification of two distinct mesenchymal stromal cell populations in human malignant glioma

**DOI:** 10.1007/s11060-016-2302-y

**Published:** 2016-10-18

**Authors:** Andreas Svensson, Tania Ramos-Moreno, Sofia Eberstål, Stefan Scheding, Johan Bengzon

**Affiliations:** 1 Lund Stem Cell Center, BMC B10, Lund University, Klinikgatan 26, 221 84 Lund, Sweden; 2 Department of Clinical Sciences, Division of Neurosurgery, Lund University, Box 117, 221 00 Lund, Sweden; 3 Department of Hematology, Skåne University Hospital, Lund, Sweden

**Keywords:** Malignant glioma, Glioblastoma, Mesenchymal stromal cell, CD90

## Abstract

Gene profiling has revealed that malignant gliomas can be divided into four distinct molecular subtypes, where tumors with a mesenchymal gene expression are correlated with short survival. The present investigation was undertaken to clarify whether human malignant gliomas contain endogenous mesenchymal stromal cells (MSC), fulfilling consensus criteria defined by The International Society for Cellular Therapy, recruited from the host. We found that MSC-like cells can be isolated from primary human malignant gliomas. Two distinct MSC-like cell populations, differing in their expression of the CD90 surface marker, were discovered after cell sorting. RNA sequencing revealed further genetic differences between these two cell populations and MSC-like cells lacking CD90 produced higher amounts of VEGF and PGE_2_ compared to cells with the true MSC phenotype, implying that the CD90^−^ MSC-like cells most probably are more active in tumor vascularization and immunosuppression than their CD90^+^ counterpart. The results highlight the CD90^−^ subpopulation as an important tumor component, however, its functional effects in glioma remains to be resolved. Using the protocols presented here, it will be possible to isolate, characterize and analyze brain tumor-derived MSC-like cells in more detail and to further test their functions in vitro and in in vivo xenograft models of glioma.

## Introduction

Mesenchymal stromal cells (MSC) are found in a variety of tissues where they constitute a stem cell pool [1]. They can be mobilized to sites of inflammation and angiogenesis, such as wounded tissue and, importantly, various solid cancers [2–4]. No single MSC-specific marker exists for in vivo detection of this cell type and it has been shown that the marker expression of MSCs greatly depends on the tissue in which they reside [5, 6]. Human MSCs are defined according to in vitro consensus criteria developed by The International Society for Cellular Therapy (ISCT); in culture, MSC should adhere to plastic, they should express CD73, CD90, CD105 but lack expression of CD11b or CD14, CD19 or CD79α, CD34, CD45 and HLA-DR, and they should be able to differentiate into adipocytes, osteoblasts and chondrocytes [7].

MSC constitute a promising vector system for cell mediated gene therapy [8] as they display inherent tumor-tropic migratory capabilities [9, 10] are found in several adult tissues [1] and are easy to isolate and expand in vitro [11]. However the use of MSCs as a tumor-tropic cellular vector system in cancer including brain tumor gene therapy also raise several concerns as it is not clarified how the cells affect the tumor and its microenvironment [12]. Consequently the presence and role of endogenous in human brain tumors has now gained attention [13]. Isolation of glioma-associated human MSCs was recently performed. Analysis of bulk cultures of primary dissociated tissue from human glioma revealed that glioma-associated MSCs were nontumorigenic stromal cells with phenotypical similarities to bone marrow-derived human MSCs [13]. Genomic sequencing of the glioma-associated MSCs suggested that most MSCs were normal non-transformed cells recruited into the glioma.

The present investigation was undertaken in order to expand on these previous recent findings. For this, a robust protocol for the detection of MSCs using 9 phenotypic markers according to the full ISCT consensus criteria has been developed. Following single cell sorting, we here report the findings of two distinct populations of glioma-derived MSC-like cells that differ in their CD90 expression, their gene expression pattern and their production of pro-angiogenic VEGF and immune-suppressive PGE_2_.

## Materials and methods

### Tumor samples

Fourteen primary brain tumor samples were obtained from the neurosurgery department at Skåne University Hospital in Lund, Sweden. Ethical permit H15 642/2008. The tissue was washed in phosphate buffered saline (PBS, Life Technologies, Carlsbad, CA, USA), placed in a Petri dish with StemMACS MSC Expansion Media (Miltenyi Biotec, Bergisch Gladbach, Germany) and minced with a scalpel. Afterwards, cells were washed in PBS and then incubated in Accutase (Sigma-Aldrich, Stockholm, Sweden) at 37 °C for 20 min. They were rotated every 5 min to prevent sedimentation. Finally, cells were passed 2–3 times through an 18 G needle before being filtered through a 100 µm nylon mesh. The single cell suspension was put in cell culture flasks with MSC Expansion Media.

### Cell culturing

Tumor-derived cells were grown adherently in MSC Expansion Media supplemented with Antibiotic–Antimycotic solution (AAS, Sigma-Aldrich) in cell culturing flasks at 37 °C and 5 % CO_2_. Half of the medium was changed 1–3 times per week. Cells at 70–100 % confluency were passaged using Accutase.

### Flow cytometry

Culture-derived cells at passage 2–4 were stained with the following monoclonal antibodies; Brilliant Violet 421-CD73 (AD2), Alexa Fluor 700-CD90 (5E10, both from Nordic BioSite, Täby, Sweden), PE-CD105 (266), PE-Cy5-HLA-ABC (G46-2.6), FITC-CD14 (M5E2), FITC-CD19 (HIB19), FITC-CD34 (581), FITC-CD45 (HI30) and FITC-HLA-DR (G46-6, all from BD Biosciences) and isotype matched controls for 30 min at 4 °C. TO-PRO-1 (Life Technologies) was used as viability marker and cells were analyzed and sorted on a FACSAria III cell sorter (BD Biosciences, Heidelberg, Germany).

### Cell sorting

Culture-derived cells analyzed on the FACSAria III cell sorter were simultaneously sorted based on surface marker expression. Two populations were sorted from each tumor, one expressing the defined MSC phenotype and one expressing the defined MSC phenotype except CD90. Cells were sorted at passage 2–4 and immediately put back in culture.

### “In vitro” differentiation

Sorted tumor-derived stromal cells at passage 8–11 were used for in vitro differentiation assays. Bone marrow-derived (BM) MSCs from healthy donors were used as positive control and the experiments were performed as described elsewhere [14].

For adipocyte differentiation, cells were cultured in StemMACS AdipoDiff Media (Miltenyi Biotec) for 14 days. They were fixed with 4 % formaldehyde solution (APL, Stockholm, Sweden) for 60 min at and then incubated in 60 % isopropanol (VWR International, Stockholm, Sweden) for 5 min before being stained with 0.18 % Oil Red O (Sigma-Aldrich) for 5 min, all at room temperature (RT).

For osteoblast differentiation, cells were cultured in osteoblast induction medium for 21 days. They were incubated in ice-cold 70 % ethanol for 1 h at 4 °C before being stained with 40 mM Alizarin Red solution (Sigma-Aldrich) for 10 min at RT.

For chondrocyte differentiation, cell pellets were grown in chondrogenesis induction medium for 28 days. They were fixed in Stefanini’s fixative [15] and then incubated in 20 % sucrose solution (Merck KGaA, Darmstadt, Germany), both at 4 °C overnight. The pellets were frozen in Tissue-Tek O.C.T. Compound (Sakura Finetek Sweden AB, Göteborg, Sweden). 5 µm thick cryosections were permeabilized and blocked with PBS supplemented with 0.3 % Triton X-100, 0.1 % sodium azide, 0.1 % fish skin gelatin (all from Sigma-Aldrich) and 10 % normal donkey serum (Jackson ImmunoResearch Europe Ltd., Suffolk, United Kingdom) for 45 min at RT. They were stained with polyclonal goat anti-human aggrecan antibody (5 µg/ml, R&D Systems) at 4 °C overnight and then Alexa Fluor 594 donkey anti-goat IgG (5 µg/ml, Life Technologies) for 60 min at RT. Nuclei were visualized by incubation with Hoechst 33342 (8.1 µM, Life Technologies) for 15 min at RT.

Stained adipocytes and osteoblasts were analyzed with a Nikon Diaphot 300 and a Nikon D70 (both from Nikon, Yurakucho, Tokyo, Japan). Chondrocyte pellets were analyzed with an Olympus BX61 and an Olympus DP72 (both from Olympus, Shinjuku, Tokyo, Japan).

### RNA-sequencing extraction and gene analysis measurements

Total RNA from GBM-47 (passage number 2); GBM-48 (passage number 2); GBM-47-derived MSC-like CD90^−^ cells (passage number 8); GBM-48-derived MSC-like CD90^−^ cells (passage number 5); GBM-47-derived MSC-like CD90^+^(passage number 10); GBM-48-derived MSC-like CD90^+^(passage number 6); U87 primary GBM cell line (passage number 30) and human bone-marrow derived MSC (hBM-MSC, passage number 1, from a 61 years old healthy male donor) was isolated by using RNAesay (Qiagen) with DNase treatment. RNA was extracted according to manufacturer instructions and their concentration was determined spectrophotometrically by Nanodrop-ND 1000 spectophotometer (Nanodrop). RNA quality was analyzed using a Bioanalyzer (Agilent) and samples with a RNA integrity greater than seven were further analyzed. Subsequent RNA amplification and analysis were largely performed as previously reported in [16, 17] except that libraries were prepared using TruSeq Stranded mRNA Kit for NeoPrep from Illumina.

### ELISA

Sorted tumor-derived stromal cells at passage 10–13 were analyzed for VEGF and PGE_2_ production using ELISA. 1 × 10^5^ cells/well were plated in a 24 well plate and grown in 1 ml MSC Expansion Media for 24 h. Supernatants were collected, centrifuged and stored at −80 °C prior to analysis. VEGF levels were determined using the Human VEGF DuoSet kit (R&D Systems). PGE_2_ levels were determined using the Prostaglandin E_2_ EIA kit (Cayman Chemical Company, Larodan Fine Chemicals, Malmö, Sweden). Experiments were performed in duplicate on three separate occasions.

### Statistical analysis

The RNA sequencing data processing was performed using the analysis pipe line method described in SCAN-B article [16]. Thus, after the first filtering processes, only the mRNAs showing differential expression between different groups are taken for further analysis. To compare the mRNA expression profile between the 2 GBM-derived MSC groups, CD90^−^ and CD90^+^ cells, simple *t* test was used and a p value <0.01 was considered significant. The VEGF and PGE_2_ production analysis was performed using Two-way ANOVA, where p < 0.05 was considered statistically significant. Linear regression analysis was performed correlating the survival time of patients to % total MSC in bulk culture or CD90^+^ cells in bulk culture or CD90^−^ cells in bulk culture in all possible combinatorial forms. A p value lower than 0.05 was considered to be significant.

## Results

### Cells with MSC marker expression profile are present in human primary brain tumor cultures

**Fig. 1:**
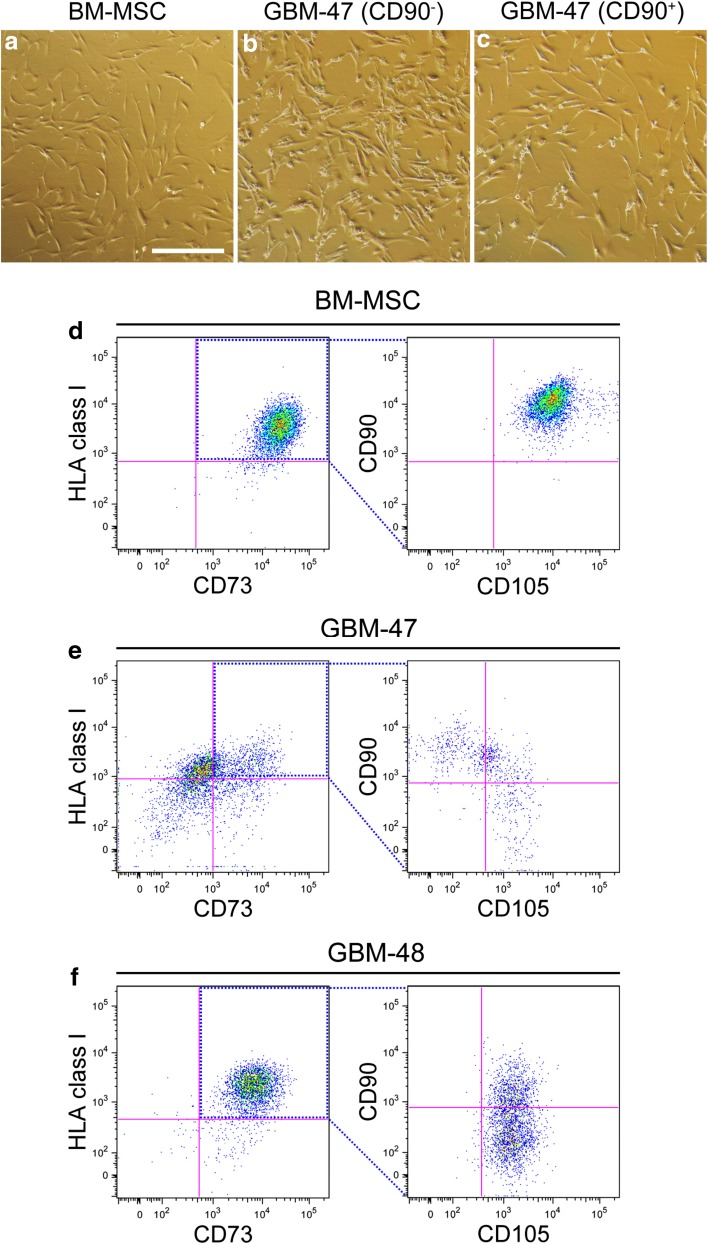
In vitro images of adherently grown **a** BM-MSCs, **b** glioma-derived MSC-like CD90^−^ cells and **c** glioma-derived MSC-like CD90^+^ cells. *Scale bar* 500 µm. **d** BM-MSCs and culture-derived tumor cells from **e** GBM-47 and **f** GBM-48 were analyzed and sorted for MSC markers with flow cytometry. Cells were stained for markers defining MSCs (CD73, CD90, CD105 and HLA class I). Doublets, dead cells and cells expressing lineage negative markers (CD14, CD19, CD34, CD45 and HLA-DR) were used as a cocktail in Lin TO-PRO-1 and have already been excluded

Tumor specimens from 14 different glioma patients grown adherently in vitro and displayed a fibroblastic morphology consistent with MSCs (Fig. 1a–c). All 14 tumor samples were grown as bulk cultures. In all of these cultures, large numbers of spindle shaped cells with a morphology fully compatible with MSCs were observed attached to the plastic surface of the culture flask. Cells in bulk cultures were easily expandable, however, since we aimed for sorting at the lowest passage number possible, bulk cultures were never passaged more than a few times. We then assessed, by flow cytometry, whether cells fulfilling the consensus marker expression profile for MSCs are present in human gliomas.

At passage 2–4, all tumors contained a small subpopulation of cells expressing the full MSC phenotype, as analysed by flow cytometry (Fig. 1d–f). Numerous cells displaying the full MSC consensus marker panel except for CD90 were detected. The fraction of MSC-like cells relative to the total number of cells in culture varied within a wide range (Table 1; Fig. 1d–f). Notable was that in the majority of the tumors, the number of cells displaying the CD90^−^ phenotype was larger than the CD90^+^ population. Routine pathological diagnosis revealed that the tumor with the noticeably highest amount of MSC-like cells was a gliosarcoma. Another interesting finding was that the low-grade astrocytoma (AC-45) contained notably fewer MSC-like cells than most of the high-grade GBMs, however no correlation was observed between patient survival and the % of MSC-like cells in the tumor (data not shown).

**Table 1:** Fourteen human brain tumors and BM-MSCs analyzed for MSC marker expression using flow cytometry

Sample	Sex	Age (years)*	Diagnosis	Grade	Passage at analysis	MSC** to total cell ratio in culture (%)	CD90^−^ MSC population in culture (%)	Survival (months)
BM-MSC	–	–	–	–	5	97.1	–	–
AC-45	m	31	Astrocytoma	II	4	0.539	3.73	24
ODG-44	f	49	Oligodendro-glioma	III	2	3.62	35.3	***
GBM-40	m	75	GBM	IV	4	13.6	85.4	9
GBM-43	m	78	GBM	IV	4	1.43	21.3	9
GBM-46	m	56	GBM	IV	3	0.417	4.03	3
GBM-47	m	58	GBM	IV	2	7.19	5.58	16
GBM-48	m	71	GBM	IV	2	29.6	63.5	1
GBM-49	f	69	GBM	IV	3	1.33	13.7	10
GBM-51	m	60	GBM	IV	2	1.68	0.525	27
GBM-52	m	76	GBM	IV	4	4.13	6.33	1
GBM-53	m	62	Gliosarcoma	IV	2	34.3	48.7	17
GBM-54	m	69	GBM	IV	2	11.0	27.4	11
GBM-55	f	62	GBM	IV	4	1.94	0.203	8
GBM-56	m	45	GBM	IV	4	0.0183	2.63	***

*Age at the time of surgery

**Positive for CD73, CD90, CD105, HLA class I; negative for CD14, CD19, CD34, CD45, HLA-DR

***Alive

### MSC phenotype-expressing cells isolated from human brain tumors can differentiate into osteoblasts and, to some extent, adipocytes and chondrocytes

Next, we determined if the cells expressing MSC markers had the capacity to differentiate into adipocytes, osteoblasts and chondrocytes. Cells were sorted using FACS according to the consensus criteria defined by ISCT. Two populations from each tumor were isolated, one expressing the defined MSC phenotype and one expressing the defined phenotype except CD90. Sorted cells from only two different tumors were able to proliferate in vitro and were thus analyzed further. Before sorting, cells had been passaged for 2–4 times and after sorting these cells were again passaged for a maximal total passage number of 14.

**Fig. 2:**
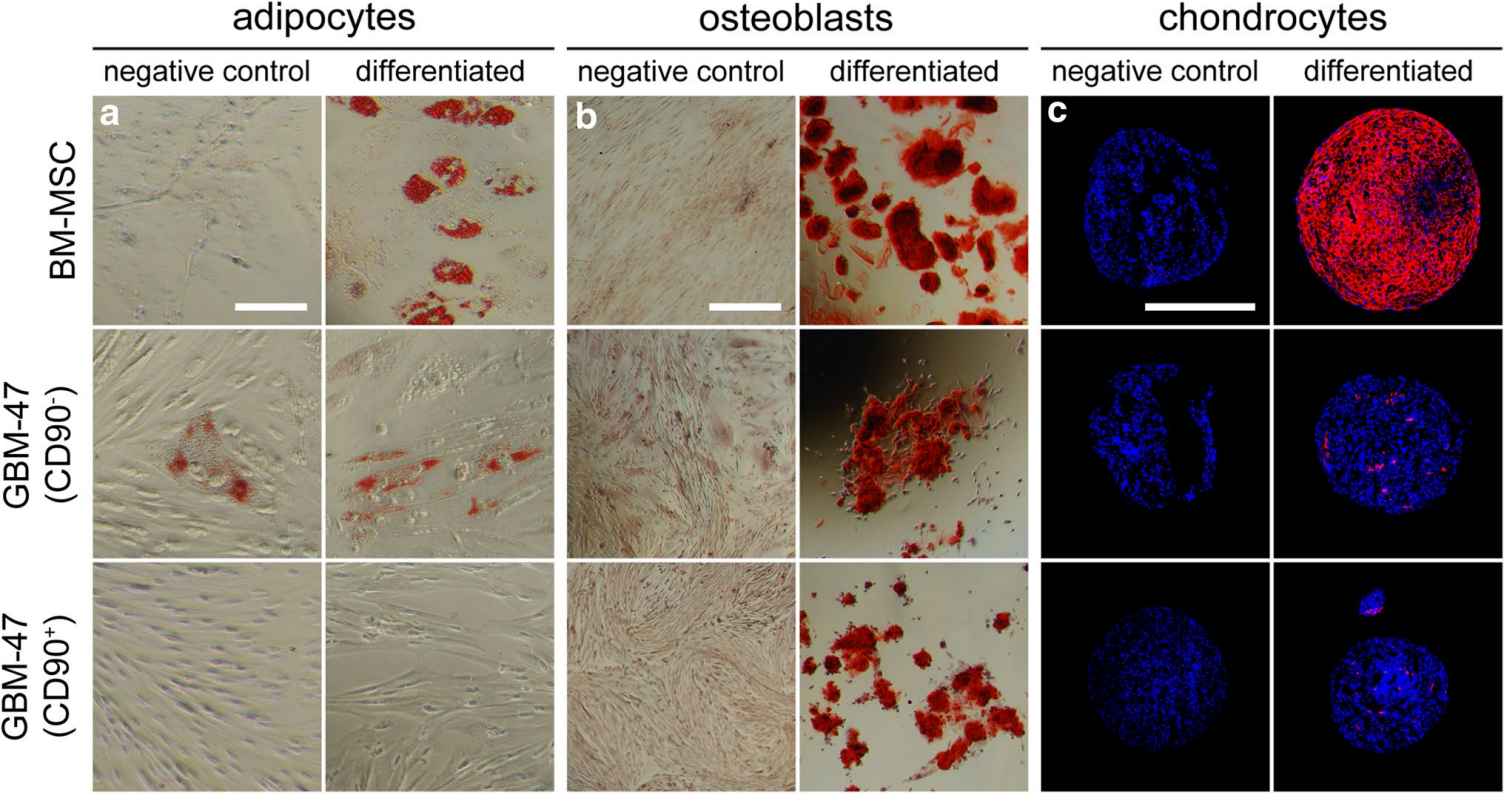
Sorted tumor-derived stromal cells were differentiated into **a** adipocytes, **b** osteoblasts and **c** chondrocytes. Light microscope images reveal lipid vacuoles in adipocytes and calcium deposits in osteoblasts. Epifluorescent images show aggrecan staining (*red*) on chondrocytes. Nuclei are blue. *Scale bar* 100 µm in **a** and 500 µm in **b** and **c**

Possible adipocyte differentiation was only detected in the CD90^−^ population of GBM-47 (Fig. 2a). In the CD90^+^ population, and in the sorted cells from GBM-48, the cells were clearly changed by the adipogenic differentiation stimulation but no clear lipid vacuole staining was visible. Further on, all sorted cells, even the ones lacking CD90, had the capacity to differentiate into osteoblasts (Fig. 2b). Finally, both the CD90^−^ and CD90^+^ population from GBM-47 formed chondrocytes (Fig. 2c), as assessed by aggrecan immunoreactivity, whereas the corresponding populations from GBM-48 only showed weak aggrecan expression (data not shown).

We conclude that cells displaying a complete MSC phenotype can be present in human brain tumor specimens. MSC-like cells lacking CD90 isolated from primary human brain tumors could differentiate into osteoblasts, adipocytes and chondrocytes, whereas the corresponding cells expressing CD90 only formed osteoblasts and chondrocytes.

### Differential mRNA expression in CD90^+^ and CD90^−^ MSC-like populations

**Fig. 3:**
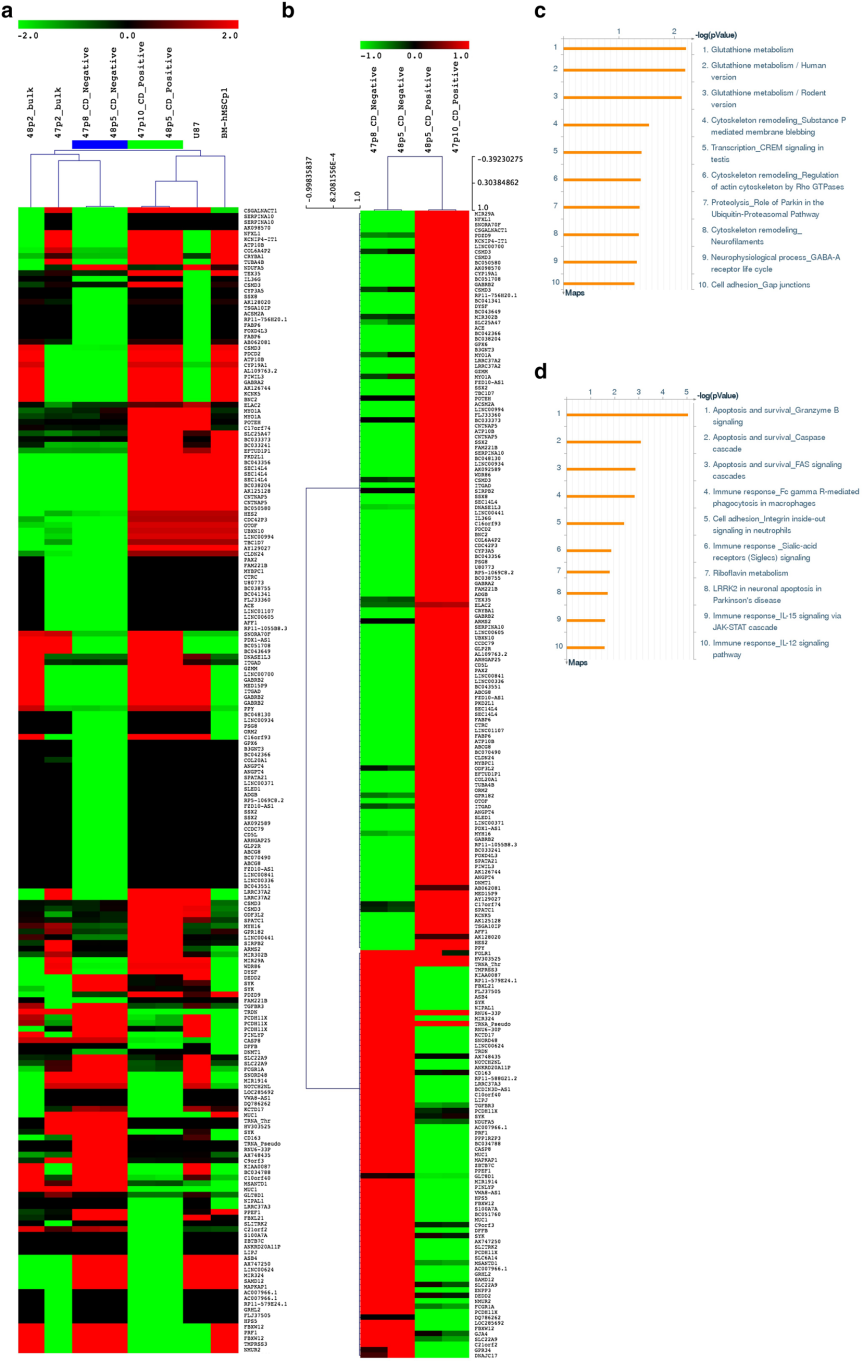
**a** Heatmap of genes differentially expressed in RNA-sequencing analysis performed on GBM-47; GBM-48; GBM-47/48-derived MSC-like CD90^−^ cells; GBM-47/48-derived MSC-like CD90^+^; U87 primary GBM cell line and bone-marrow derived MSC show glioma-derived MSC-like CD90^−^ and CD90^+^ cells gene expression patterns to be different (p < 0.01, paired Student’s *t* test). CD90^+^ cells clustering with U87 and later with hBM-MSC, may possibly reflect a similar pluripotency stage. **b** Unsupervised hierarchical sample clustering between glioma-derived MSC-like CD90^−^ and CD90^+^ cells (p < 0.01, paired Student’s *t* test). **c** Significantly up-regulated canonical pathways within the transcriptional profile from the MSC-like CD90^+^ cells versus MSC-like CD90^−^ cells. **d** Significantly down-regulated canonical pathways within the transcriptional profile from the MSC-like CD90^+^ cells versus MSC-like CD90^−^ cells

Differentially expressed gene (DEG) analysis of the transcriptional profile associated with the different cell types here analyzed (GBM, GBM-derived MSC, U87 and hBM-MSC) revealed that there is a different mRNA expression profile between the 2 sorted MSC-like cells, CD90^+^ and CD90^−^ (Fig. 3a). A *t* test was subsequently performed to identify differentially expressed genes between CD90^−^ and CD90^+^ cell types, this resulted in total, 211 genes (135 up regulated and 76 down regulated in CD90^+^ cell lines). Differentially expressed genes were next subjected to functional annotation analysis using Metacore web-based software (https://portal.genego.com/) and results show that, amongst the up regulated genes in the CD90^+^ cell lines, 50 % were enriched for the glutathione metabolism; 25 % were enriched for the cytoskeleton remodeling and 8.3 % for cell adhesion. Amongst the down-regulated genes, 46.7 % were involved in apoptosis and survival; 33.3 % were involved in the immune response and 13.3 % in cell adhesion (Fig. 3b–d). Interestingly, and although cell surface expression of CD90 clearly separates two distinct populations of glioma-derived MSC-like cells, the CD90 mRNA expression profile was not significantly different between these two cell populations and thus, was not taken into account for the different analysis (Fig. 3).

### The CD90^−^ MSC-like population produces more VEGF and PGE_2_ compared to its CD90^+^ counterpart

**Fig. 4:**
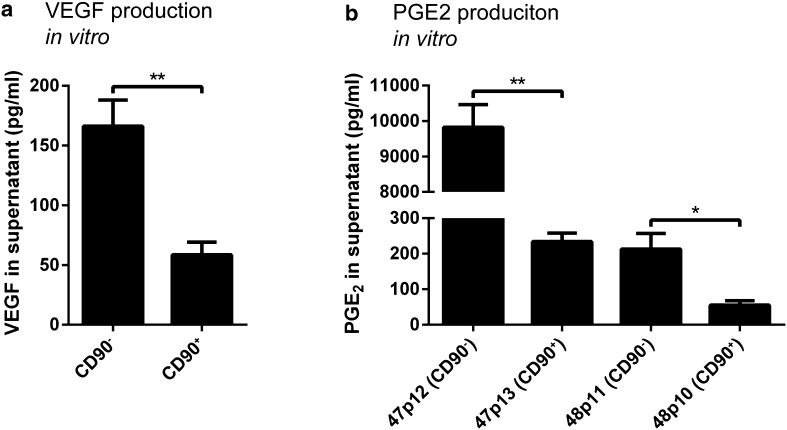
**a** VEGF and **b** PGE_2_ production in vitro by two different cell populations from each of two GBMs. The CD90^−^ populations from GBM-47 and GBM-48 produce higher levels of both VEGF and PGE_2_ compared to the CD90^+^ populations from the same tumors. 1 × 10^5^ cells were grown in 1 ml MSC Expansion Media for 24 h and the supernatants were analyzed with ELISA. Each experiment was performed three times in duplicate and VEGF data from both tumors was pooled together

The two cell populations that were expandable following cell sorting were analyzed for VEGF and PGE_2_ production. The results show that the CD90^−^ cells from two GBMs secreted significantly higher levels of VEGF compared to the CD90^+^ cells from the same tumors (mean 166 and 58.6 pg/ml, respectively, p < 0.01; Fig. 4a).

The CD90^−^ population from GBM-47 also produced considerably more PGE_2_ compared to the CD90^+^ population from the same tumor (mean 9830 and 234 pg/ml, respectively, p < 0.01). Similar, the PGE_2_ production of the CD90^−^ population from GBM-48 produced significantly more PGE_2_ compared to the CD90^+^ population from the same tumor (mean 213 and 55.5 pg/ml, respectively, p < 0.05; Fig. 4b).

## Discussion

Malignant gliomas with a mesenchymal gene expression profile are associated with an invasive growth pattern and short survival and recurring tumors often display a mesenchymal phenotype [18, 19]. In this study, we analyzed 14 primary human malignant gliomas and conclude that all of them harbor cells expressing surface markers defined by ISCT [7].

By employing a stricter flow cytometric analysis, adhering to the ISCT criteria, and by, for the first time for this particular purpose, sorting cells, we expand on previous reports of MSC-like cells in malignant glioma [13]. We found that malignant gliomas harbor two distinct MSC-like cell populations, differing in their CD90 expression. Although a different tri-mesenchymal differentiation capacity was noticed between CD90^+^ and CD90^−^ populations, as indicated by adipocyte differentiation only in cells lacking CD90, this data is preliminary and further experiments, optimizing the expansion of MSC clones from a larger number of tumor samples, is warranted in order to confirm if the absence of adipocyte differentiation is a distinctive feature of CD90^+^ cell population.

In most tumor samples, the CD90^−^ population was larger than the CD90^+^ population. Both populations were expandable and retained their proliferative capacity in vitro. Unfortunately, these cell populations were difficult to detach from the plastic surface during the first passages and we could only perform complete studies in cells derived from 2 patients. The number of MSC-like cells compared to the total number of cells (MSC to total cell number in culture in Table 1) following 2–4 passages in vitro showed a very wide variation. Yet, the present study was not designed in order to quantify the ratio of MSC-like cells within glioma stroma. Interestingly though, the tumor with the highest proportion of MSC-like cells was a gliosarcoma, a GBM subtype with a clear mesenchymal component [14].

To confirm that the two MSC-like cell populations CD90^+^ and CD90^−^ constitute two distinct cell populations, we analyzed their gene expression profile. Although we were limited in the number of samples, the mRNA expression profiles clearly support the notion that these are, in fact, two different populations. Intriguingly, although the cell surface expression of CD90 separates these populations, their CD90 mRNA expression profile did not differ, suggesting that cell surface glycoprotein CD90 could be either engulfed from the surface due to a cell reaction to some environmental stimuli, thus becoming undetectable on the surface or, that CD90^−^ cells react to some stimuli and overexpress CD90 as a response to the stimuli. In vitro angiogenic stimuli, for example, generate hM-MSC lacking CD90 expression [20] whilst overexpression of surface cell markers upon inflammatory stimuli has been already reported for neurotrophic receptors [21]. In addition, mechanical stress has been reported to decrease CD90 cell surface glycoprotein in MSC [22]. Since little mechanical stress was performed during culturing, and the number of passages were low when mRNA expression analysis was performed, this hypothesis is quite unlikely. Alternatively, the CD90 mRNA could suffer any type of posttranslational preventing the receptor from being visible on the surface [23, 24]. Future experiments for a further deep characterization of CD90^+^ and CD90^−^ populations are envisaged.

Likewise, the functional significance of CD90 expression, or lack thereof, on glioma-derived MSC-like cells needs further investigations. Previous studies have suggested that CD90^+^ pericytes within gliomas promote vascularization and immunosuppression [15]. However, CD90 has also been reported to be a tumor suppressor [25]. The present study might lend some support to the latter, as we found that the MSC-like CD90^−^ cells could be producing elevated levels of VEGF compared to the CD90^+^ cells from the same tumors. If confirmed, this could indicate that the CD90^−^ population of glioma-derived MSC-like cells might be more actively involved in tumor angiogenesis than the CD90^+^ subpopulation. In addition to its critical role in tumor angiogenesis, VEGF has been reported to attract MSCs themselves to glioma cells [3]. Furthermore, VEGF has been reported to stimulate MSC proliferation [26], indicating that autocrine mechanisms might function to stimulate MSC recruitment and proliferation within glioma.

The immunosuppressive function of BM-MSCs is well characterized and utilized clinically [27]. Among the key immunoactive factors expressed by MSCs is prostaglandin E_2_ (PGE_2_) [28], which has been shown to induce immunosuppression within tumors [15, 29]. Because of technical reasons, we were only able to expand FACS sorted cells from two glioblastoma, which severely restricted their further analysis. However, interestingly, the CD90^−^ MSC-like cells secreted markedly higher amounts of PGE_2_ compared to the CD90^+^ cells. PGE_2_ is a molecule known to be important for BM-MSC-induced immunosuppression [30], suggesting that glioma-derived MSC-like cells, and the CD90^−^ subpopulation in particular, may actively contribute to the glioma immunosuppression.

Even though the source of the glioma-derived MSC-like cells is unknown, the most probable explanation is that the tumor recruits them from normal tissue. Inflammatory factors, such as IL-8 [31], monocyte chemotactic protein-1 [32] and stromal cell-derived factor 1α [33] are known to be expressed in gliomas and have been reported as attractants of MSCs in other cancers [34, 35]. Further on, the extensive angiogenesis within malignant gliomas is believed to play a major part in MSC migration. In addition to VEGF, angiogenic factors such as platelet-derived growth factor-BB [36] and transforming growth factor-β1 [3], known to be involved in glioma angiogenesis, have been shown to mediate MSC recruitment [3, 34]. Moreover, we have previously shown that drug-mediated angiogenesis inhibition markedly decreased the migration of BM-MSCs transplanted into experimental rat gliomas [10].

In conclusion, we show that two distinct MSC-like cell populations, differing in their expression of the CD90 surface marker expression, can be isolated from primary human malignant gliomas. Further analysis revealed important differences between these two populations with regards to mRNA expression patterns and, present results show that CD90^−^ cells produced higher amounts of VEGF and PGE_2_ compared to cells with the true MSC phenotype, implying that CD90^−^ cells may be more active in tumor vascularization and immunosuppression than their CD90^+^ counterpart. Taken together, our results highlight the CD90^−^ MSC-like subpopulation as an important tumor component, however, its functional effects in glioma remains to be resolved. Using the protocols presented here, it will be possible to isolate, characterize and analyze brain tumor-derived MSC-like cells in more detail and to further test their functions in vitro and in in vivo xenograft models of glioma.
